# Does infection with human immunodeficiency virus have any impact on the cardiovascular outcomes following percutaneous coronary intervention?: a systematic review and meta-analysis

**DOI:** 10.1186/s12872-017-0624-0

**Published:** 2017-07-17

**Authors:** Pravesh Kumar Bundhun, Manish Pursun, Wei-Qiang Huang

**Affiliations:** 1 Institute of Cardiovascular Diseases, the First Affiliated Hospital of Guangxi Medical University, Nanning, Guangxi 530027 People’s Republic of China; 2 Guangxi Medical University, Nanning, Guangxi 530027 People’s Republic of China; 3 Institute of Cardiovascular Diseases, the First Affiliated Hospital of Guangxi Medical University, Nanning, Guangxi 530021 People’s Republic of China

**Keywords:** Human immunodeficiency virus, Percutaneous coronary intervention, Cardiovascular outcomes, Acquired immune deficiency syndrome, Coronary artery disease, Highly active antiretroviral therapy

## Abstract

**Background:**

A direct link between human immunodeficiency virus (HIV)-infected patients and the risk of cardiovascular diseases (CVD) has been shown in recent scientific research. However, this issue is controversial since other previous reports showed no apparent impact of HIV or its anti-retroviral drugs on the cardiovascular system. We aimed to systematically compare the postinterventional adverse cardiovascular outcomes which were observed in patients with and without HIV infection during a mean follow up period ranging from 1 year to 3 years.

**Methods:**

Common electronic databases were searched for studies which compared postinterventional adverse cardiovascular outcomes [mortality, myocardial infarction (MI), cardiac death, target vessel revascularization (TVR), target lesion revascularization (TLR), stroke and major adverse cardiac events (MACEs)] in patients with and without HIV infection. Statistical analysis was carried out by the RevMan 5.3 software whereby Odds Ratios (OR) and 95% Confidence Intervals (CIs) were generated.

**Results:**

Two thousand two hundred and sixty-eight (2268) patients (821 patients were HIV positive and 1147 patients were HIV negative) were analyzed. The current results showed that mortality was not significantly increased among patients who were HIV positive with OR: 1.13, 95% CI: 0.65–1.96; *P* = 0.66. Cardiac death was also similarly reported with OR: 1.16, 95% CI: 0.50–2.68; *P* = 0.74. However, even if recurrent MI, TVR, TLR, MACEs and stroke were higher in patients who were HIV positive, with OR: 1.32, 95% CI: 0.88–2.12; *P* = 0.18, OR: 1.36, 95% CI: 0.88–2.12; *P* = 0.17, OR: 1.22, 95% CI: 0.72–2.06; *P* = 0.46, OR: 1.29, 95% CI: 0.89–1.85; *P* = 0.17 and OR: 1.47, 95% CI: 0.44–4.89; *P* = 0.53 respectively, these results were not statistically significant.

**Conclusion:**

Patients who were infected with HIV had similar mortality post coronary intervention compared to patients who were not infected by the virus, during a mean follow-up period of 1–3 years. In addition, no significant increase in MI, TVR, TLR, MACEs and stroke were observed during this follow up period. Therefore, it might be concluded that no apparent impact of HIV on the cardiovascular outcomes was observed post coronary intervention.

## Background

In its earlier stage known as Human Immunodeficiency Virus (HIV) infection and later manifesting as Acquired Immune Deficiency Syndrome (AIDS), HIV/AIDS has been in the headlines since the year 1981. For many years, Highly Active Anti-Retroviral Therapy (HAART) has been the main treatment expected to have prolonged survival and improved quality of life in these HIV infected patients [1].

A clear link between HIV-infected patients and the risk of cardiovascular diseases (CVD) has been shown in recent scientific research [2]. The PREmature VAscular LEsions and Antiretroviral Therapy (PREVALEAT II) Cohort further supported this point [3].

However, the use of HAART and cardiovascular manifestations in HIV infected patients has mainly been reported in case studies [4] and compared to infected patients who were not treated by HAART therapy, an increase in the yearly incidence of Myocardial Infarction (MI) was observed among patients who were treated with HAART [5].

Nevertheless, this issue is still controversial. Even if only a minority of research has been carried out on this particular topic, a few studies showed no impact of HIV or its anti-retroviral therapy on the cardiovascular system [6].

Percutaneous Coronary Intervention (PCI) is the most common invasive procedure which is being carried out in this new Era. Any factor which affects the cardiovascular system should probably influence cardiovascular outcomes following PCI. Therefore, this study aimed to systematically compare the postinterventional adverse cardiovascular outcomes which were observed in patients with and without HIV infection during a mean follow up period of 1 to 3 years.

## Methods

### Data sources and search strategy

Online electronic databases [PubMed/Medline, EMBASE (www.sciencedirect.com) and the Cochrane databases] were searched for Randomized Controlled Trials (RCTs) and Observational studies (which were published in English language) comparing postinterventional adverse cardiovascular outcomes which were reported in patients with and without HIV infection.

The following searched terms were used:

1. Human Immunodeficiency Virus and Percutaneous Coronary Intervention;
2. HIV and PCI;
3. Acquired Immune Deficiency Syndrome/AIDS and PCI;
4. Coronary angioplasty and Human Immunodeficiency Virus;
5. Acute coronary syndrome and Human Immunodeficiency Virus;
6. Coronary revascularization and Human Immunodeficiency Virus;
7. Percutaneous revascularization and Human Immunodeficiency Virus;
8. Coronary stenting and Human Immunodeficiency Virus.

### Inclusion and exclusion criteria

Studies were included if:

- They were RCTs or observational studies which compared post-interventional adverse cardiovascular outcomes [mortality, myocardial infarction (MI), cardiac death, target vessel revascularization (TVR), target lesion revascularization (TLR), stroke and major adverse cardiac events (MACEs)] in patients with and without HIV infections.

Studies were excluded if:

- They were meta-analyses, or case studies.
- They did not report the above-mentioned adverse cardiovascular outcomes as their clinical endpoints.
- They did not involve patients with HIV infections and their respective controls.
- They did not involve patients who underwent PCI.

### Definitions, outcomes and follow up periods

The endpoints which were considered relevant in this analysis included:

- All-cause mortality
- Cardiac mortality
- Recurrent MI
- TVR
- TLR
- Stroke
- MACEs (death, MI and revascularization). One study reported major adverse cardiovascular and cerebrovascular events (MACCEs). Therefore, we combined MACEs and MACCEs together during the analysis.

This analysis had a follow up period of 1 to 3 years. The outcomes which were reported and their corresponding follow up periods were summarized in Table 1.

**Table 1:** Reported Outcomes

Study	Reported outcomes	Follow up periods
Badr 2015 [10]	Death, cardiac death, TLR, TVR, MI, stroke, MACEs	24 months
Boccara 2006 [11]	TVR, MACEs	20 months
Boccara 2011 [12]	MACCEs, cardiac death, stroke, TLR, TVR	12 months
Lorgis 2013 [13]	MI, stroke, death	12 months
Matetzky 2003 [14]	MI, death, cardiac death	14 months
Ren 2009 [15]	MACEs, death, cardiac death, MI, TVR, TLR	37 months

*Abbreviations*: *MI* myocardial infarction, *TLR* target lesion revascularization, *TVR* target vessel revascularization, *MACEs* major adverse cardiac events, *MACCEs* major adverse cardiac and cerebrovascular events

### Data extraction and review

Two authors (PKB and MP) carefully reviewed the studies and the data which were reported. Information concerning the author names, the year of patients’ enrollment, the total number of HIV positive and HIV negative patients respectively, the outcomes and follow up periods which were reported as well as the baseline features were carefully extracted from the selected studies. During this data extraction process, any disagreement which was encountered by any of these two authors was discussed and resolved by the third author (WQH)).

### Statistical analysis

Reporting guideline which was used: The Preferred Reporting Items for Systematic Reviews and Meta-Analyses [7].

Since this is a meta-analysis of several studies, inconsistency across these studies were possible [8]. Therefore, assessment of heterogeneity was carried out to provide relevant results. Heterogeneity across the studies were assessed by first of all, the Cochrane Q statistic test (*P* value less or equal to 0.05 was considered statistically significant) and secondly the I^2^ statistic test (high I^2^ value = increased heterogeneity, lower I^2^ value = low heterogeneity).

If I^2^ was greater than 50%, a random effects model was used during the analysis, whereas if I^2^ was less than 50%, a fixed effects model was used.

The latest version of RevMan (version 5.3) was used to carry out this analysis [9], whereby odds ratios (OR) with 95% confidence intervals (CI) were used as the statistical parameters.

Funnel plot was visually assessed for evidence of publication bias.

Ethical committee or medical institutional board approval was not required.

## Results

### Search result

One hundred and six (106) articles were searched from the electronic databases. After a careful screening of the abstracts and titles, eighty-one (81) articles were eliminated since they were not relevant.

Among the remaining twenty-five (25) articles, eight (8) articles were duplicates and were spontaneously eliminated.

Seventeen (17) full-text articles were assessed for eligibility.

**Fig. 1:**
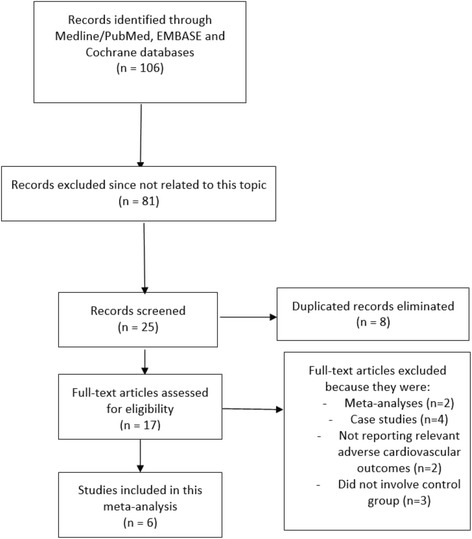
Flow diagram representing the study selection

Eleven (11) full text articles were eliminated since:

- four (4) publications were case studies,
- two (2) publications were meta-analyses,
- two (2) articles did not report the relevant adverse cardiovascular outcomes,
- three (3) articles did not involve any control group.
  Finally, six (6) articles [10–15] were selected and included in this systematic review and meta-analysis (Fig. 1).

### General features of the studies which were included

Table 2 represents the general features of the studies which were included in this systematic review and meta-analysis.

**Table 2:** General features of the studies which were included

Studies	Patients’ enrollment	No of HIV positive patients (*n*)	No of HIV negative patients (*n*)	Total no of patients (*n*)
Badr 2015 [10]	2003–2011	112	112	244
Boccara 2006 [11]	2001–2003	50	50	100
Boccara 2011 [12]	2003–2006	103	195	298
Lorgis 2013 [13]	2005–2009	435	945	1380
Matetzky 2003[14]	1998–2000	24	48	72
Ren2009 [15]	2000–2007	97	97	194
Total (*n*)		821	1447	2268

*Abbreviations*: *HIV* human immunodeficiency virus

Two thousand two hundred and sixty-eight (2268) patients were analyzed. Eight hundred and twenty-one (821) patients were HIV positive whereas 1147 patients were HIV negative. According to Table 2, patients’ enrollment period ranged from the year 1998 to the year 2011. Study Lorgis2013 consisted of the highest number of patients among all the other studies which were included in this analysis.

The quality assessment of the studies was carried out by the Newcastle Ottawa Scale (NOS) [16] whereby grades were allotted according to a star system in order to rate the methodological qualities of these studies. NOS consisted of eight items, which were classified in three different groups namely selection, comparability, and outcome or exposure. A maximum total number of nine stars (*********) were possible.

The methodological quality of each study was assessed and graded as followed:

Badr2015: ******* (7 stars).

Boccara2006: ****** (6 stars).

Boccara2011: ******** (8 stars).

Lorgis2013: ******** (8 stars).

Matetzky2003: ***** (5 stars).

Ren2009: ******* (7 stars).

### Baseline features of the participants

The baseline characteristics of the participants were represented in Table 3.

**Table 3:** Baseline features of the participants

Study	Mean age (yrs)	Males (%)	HT (%)	CS (%)	DM (%)
	HIV/Non-HIV	HIV/Non-HIV	HIV/Non-HIV	HIV/Non-HIV	HIV/Non-HIV
Badr 2015 [10]	58.0/58.0	64.3/64.3	84.8/83.0	30.4/26.8	12.7/12.7
Boccara 2006 [11]	43.3/44.0	90.0/88.0	-	-	-
Boccara 2011 [12]	48.0/50.0	93.0/94.0	18.0/24.0	59.0/64.0	9.0/12.0
Lorgis 2013 [13]	50.0/68.3	88.6/66.3	17.4/35.9	29.6/14.2	9.1/18.2
Matetzky 2003 [14]	47.0/48.0	88.0/88.0	29.0/44.0	58.0/48.0	12.0/19.0
Ren 2009 [15]	53.0/54.0	100/100	46.0/67.0	24.0/26.0	10.0/26.0

*Abbreviations*: *yrs.* years, *HIV* human immunodeficiency virus, *HT* hypertension, *CS* current smoker, *DM* diabetes mellitus

According to Table 3, (43.3–68.3 years) was the mean age of the participants. Three studies, namely studies Boccara 2006, Matetzky 2003 and Boccara 2011 consisted of younger patients in comparison to the other studies. Study Ren 2009 consisted only of male patients. Study Badr 2015 consisted of a larger number of patients with hypertension. Only a small percentage of patients suffered from diabetes mellitus. According to Table 3, almost no significant difference was observed in the baseline characteristics of the participants within the two groups.

### Analysis of the adverse cardiovascular outcomes

The postinterventional adverse cardiovascular outcomes which were observed in patients with and without HIV infection were represented in Table 4.

**Table 4:** Results of this meta-analysis

Outcomes analyzed	OR with 95% CI	*P* value	I^2^ (%)	Fixed or random effects model used
Mortality	1.13 [0.65–1.96]	0.66	0	Fixed
Cardiac death	1.16 [0.50–2.68]	0.74	0	Fixed
Recurrent MI	1.32 [0.88–2.12]	0.18	28	Fixed
TVR	1.36 [0.88–2.12]	0.17	0	Fixed
TLR	1.22 [0.72–2.06]	0.46	0	Fixed
MACEs	1.29 [0.89–1.85]	0.17	0	Fixed
Stroke	1.47 [0.44–4.89]	0.53	0	Fixed

*Abbreviations*: *MI* myocardial infarction, *TVR* target vessel revascularization, *TLR* target lesion revascularization, *MACEs* major adverse cardiac events, *OR* odds ratios, *CI* confidence intervals

Current results showed that, during a mean follow up period of 1–3.1 years, mortality was not significantly increased in the HIV positive group with OR: 1.13, 95% CI: 0.65–1.96; *P* = 0.66. Cardiac death was also noted to be similar with OR: 1.16, 95% CI: 0.50–2.68; *P* = 0.74.

**Fig. 2:**
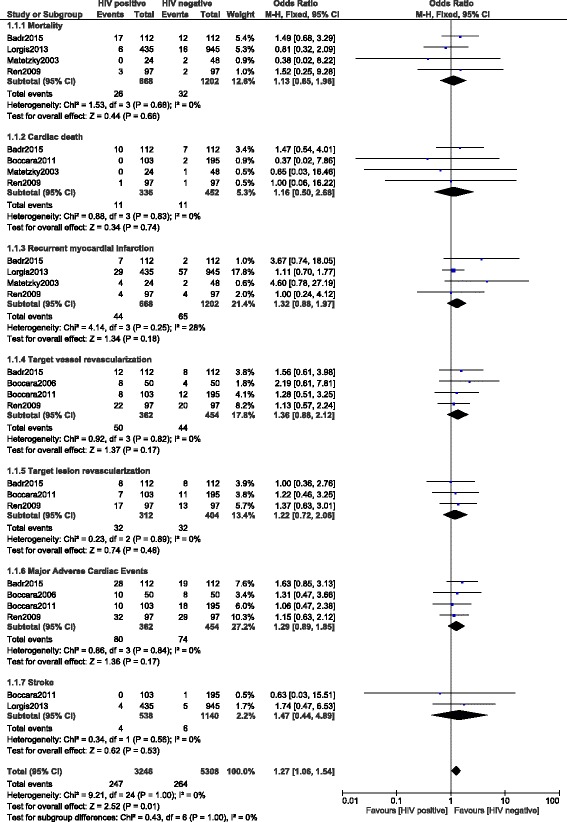
Postinterventional Adverse Cardiovascular Outcomes which were observed between HIV positive and HIV negative patients

However, even if the other adverse cardiovascular outcomes including recurrent MI, TVR, TLR, MACEs and stroke favored HIV negative patients with OR: 1.32, 95% CI: 0.88–2.12; *P* = 0.18, OR: 1.36, 95% CI: 0.88–2.12; *P* = 0.17, OR: 1.22, 95% CI: 0.72–2.06; *P* = 0.46, OR: 1.29, 95% CI: 0.89–1.85; *P* = 0.17 and OR: 1.47, 95% CI: 0.44–4.89; *P* = 0.53 respectively, the results were not statistically significant in this analysis. These results have been summarized in Table 4 and illustrated in Fig. 2.

### Sensitivity analysis

Six observational studies were included in this analysis. Therefore, to make sure that the result of this analysis was not influenced by any of the studies, we carried out a sensitivity analysis (one by one exclusion of studies with re-assessment of the results). Excluding study Badr2015 did not affect the main result of this analysis with OR: 0.86, 95% CI: 0.39–1.90; *P* = 0.71 for mortality, OR: 0.61, 95% CI: 0.11–3.32; *P* = 0.57 for cardiac death, OR: 1.20, 95% CI: 0.79–1.83; *P* = 0.39 for recurrent MI, OR: 1.31, 95% CI: 0.79–2.16; *P* = 0.29 for TVR, OR: 1.31, 95% CI: 0.71–2.42; *P* = 0.38 for TLR and OR: 1.15, 95% CI: 0.74–1.79; *P* = 0.53 for MACEs. There was no significant difference in outcomes.

When study Matetzky2003 was excluded and a new analysis was carried out, still no significant difference in results were obtained with OR: 1.19, 95% CI: 0.68–2.08; *P* = 0.55 for mortality, OR: 1.21, 95% CI: 0.50–2.91; *P* = 0.67 for cardiac death and OR: 1.22, 95% CI: 0.81–1.85; *P* = 0.34 for recurrent MI.

When study Ren2009 was excluded, still no significant difference was observed in the results with OR: 1.10, 95% CI: 0.62–1.96; *P* = 0.75 for mortality, OR: 1.17, 95% CI: 0.49–2.83; *P* = 0.72 for cardiac death, OR: 1.35, 95% CI: 0.89–2.05; *P* = 0.16 for recurrent MI, OR: 1.56, 95% CI: 0.87–2.79; *P* = 0.13 for TVR, OR: 1.11, 95% CI: 0.55–2.25; *P* = 0.78 for TLR and OR: 1.37, 95% CI: 0.87–2.15; *P* = 0.18 for MACEs.

When the remaining studies were excluded and a new analysis was carried out, still no significant difference in results was obtained as shown in Table 5, implying that for all of the above analyses, sensitivity analyses yielded consistent results.

**Table 5:** Sensitivity analysis showing odds ratios, with 95% confidence interval, the *P* value and the I^2^ value

Study which was excluded	Mortality	Cardiac death	Recurrent MI	TVR	TLR	MACEs
Badr 2015 [10]	0.86 [0.39–1.90], *P* = 0.71, I^2^ = 0%	0.61 [0.11–3.32], *P* = 0.57, I^2^ = 0%	1.20 [0.79–1.83], *P* = 0.39, I^2^ = 15%	1.31 [0.79–2.16], *P* = 0.29, I^2^ = 0%	1.31 [0.71–2.42], *P* = 0.38, I^2^ = 0%	1.15 [0.74–1.79], *P* = 0.53, I^2^ = 0%
Boccara 2006 [11]	**-**	**-**	**-**	1.27 [0.79–2.04], *P* = 0.32, I^2^ = 0%	-	1.28 [0.87–1.89], *P* = 0.21, I^2^ = 0%
Boccara 2011 [12]	**-**	1.32 [0.54–3.23], *P* = 0.55, I^2^ = 0%	-	1.39 [0.84–2.29], *P* = 0.20, I^2^ = 0%	1.22 [0.66–2.27], *P* = 0.53, I^2^ = 0%	1.35 [0.90–2.03], *P* = 0.15, I^2^ = 0%
Lorgis 2013 [13]	1.36 [0.68–2.72], *P* = 0.38, I^2^ = 0%	-	2.32 [0.96–5.58], *P* = 0.06, I^2^ = 11%	**-**	**-**	**-**
Matetzky 2003 [14]	1.19 [0.68–2.08], *P* = 0.55, I^2^ = 0%	1.21 [0.50–2.91], *P* = 0.67, I^2^ = 0%	1.22 [0.81–1.85], *P* = 0.34, I^2^ = 3%	-	**-**	**-**
Ren 2009 [15]	1.10 [0.62–1.96], *P* = 0.75, I^2^ = 0%	1.17 [0.49–2.83], *P* = 0.72, I^2^ = 0%	1.35 [0.89–2.05], *P* = 0.16, I^2^ = 50%	1.56 [0.87–2.79], *P* = 0.13, I^2^ = 0%	1.11 [0.55–2.25], *P* = 0.78, I2 = 0%	1.37 [0.87–2.15], *P* = 0.18, I^2^ = 0%

*Abbreviations*: *MI* myocardial infarction, *TVR* target vessel revascularization, *TLR* target lesion revascularization, *MACEs* major adverse cardiac events

**Fig. 3:**
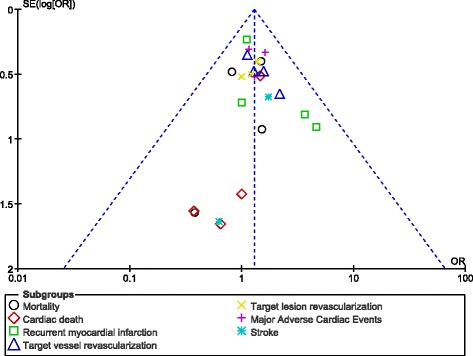
Funnel plot showing publication bias

Also, almost no evidence of publication bias was observed (Fig. 3).

## Discussion

This study aimed to compare the postinterventional adverse cardiovascular outcomes which were observed in patients with and without HIV infection.

Current results showed no significant difference in cardiovascular outcomes among patients who were or were not infected by HIV during a follow up period of 1 to 3 years post- PCI.

Previous studies showed a moderate contribution of HIV virus and HAART therapy in the pathogenesis and development of atherosclerosis in infected patients further accelerating coronary artery disease (CAD). Recent observational studies involving larger number of HIV-infected patients showed an increase incidence of MI in association with a longer exposure to antiretroviral therapies [17]. Dysfunction of the endothelium, and premature atherosclerosis have been observed among HIV positive patients who were treated by HAART whose mechanism is considered to affect lipid alteration and inflammatory mechanisms contributing to premature atherosclerosis [18]. Studies have also shown AIDS virus to contribute to these effects by inducing inflammatory reactions intracellularly. HIV virus is able to penetrate coronary artery endothelial cell membrane and induce inflammatory and intracellular responses and reactions further activating endothelial cells [19].

A study carried out by Boccara et al. consisted of data that suggested the presence of an accelerated process of coronary atherosclerosis in HIV positive due to factors including a higher prevalence of conventional and emerging risk factors such as chronic inflammation and immune activation and the role of antiretroviral therapy [20].

Similar to the current analysis, the single-centered study involving patients from MedStar Washington Hospital showed similar short and long-term outcomes reported between HIV positive and HIV negative patients who underwent PCI with drug eluting stents. Moreover, the retrospective study of prospectively collected cohorts investigating the clinical presentation of ACS in HIV infected adults showed a similar prognosis between HIV positive and HIV negative patients [21]. Another study showing the outcomes of patients with HIV undergoing cardiac surgery in the United States showed no increase in mortality among patients with HIV infection compared to those patients not infected by the virus [22].

However, results from the study by Hsue et al. involving data from San Francisco General Hospital unexpectedly showed a higher rate of restenosis after PCI in patients who were positive for HIV virus [23], which was completely different from the current results. But their study involved HIV positive patients with a low level of high density lipoproteins.

A systematic review and meta-analysis investigating acute coronary syndrome (ACS) in HIV positive patients showed HIV infected patients who were admitted due to ACS faced a substantial risk of short term death, and a significant long term risk of MI and revascularization, especially in those patients receiving protease inhibitors [24]. However, our analysis, which involved a larger number of patients, showed a completely different result from that study.

In addition, registry data involving twelve sites in Europe, South Africa and the United States also showed HIV patients suffering from ACS to have a significantly increased risk for cardiovascular death if specific treatments with nucleoside-reverse transcriptase inhibitors were not started [25]. The study also showed a significantly higher risk of MI with a low CD4 cell count (<200 cells/mm^3^) indicating that HIV/AIDS with lower CD4 cell counts might further contribute to cardiovascular complications.

This analysis satisfied almost all the criteria to be considered solid enough for a meta-analysis. Despite the inclusion of observational studies, a very low level of heterogeneity was surprisingly reported among all the subgroups assessing the different clinical outcomes which should be considered a strong point of this analysis.

This analysis is also among the first meta-analyses comparing postinterventional adverse cardiovascular outcomes in patients with and without HIV infection. Moreover, it consisted of a larger number of patients (both HIV positive and HIV negative). The low level of heterogeneity reported among all the subgroups should further add up to its novelty.

However, due to a limited number of HIV positive patients, the analysis might not generate robust results. In addition, this analysis only involved data which were obtained from observational studies which are not considered as good as data which are found in RCTs. Moreover, all the studies did not report similar cardiovascular outcomes. Therefore, when the outcomes were analyzed, not all of the studies were involved each time.

## Conclusion

Patients who were infected with HIV had similar mortality post coronary intervention compared to patients who were not infected by the virus, during a mean follow-up period of 1–3 years. In addition, no significant increase in MI, TVR, TLR, MACEs and stroke were observed during this follow up period. Therefore, it might be concluded that no apparent impact of HIV on the cardiovascular outcomes was observed post coronary intervention.

## Abbreviations

AIDS: acquired immune deficiency syndrome
CAD: coronary artery disease
HAART: highly active antiretroviral therapy
HIV: human immunodeficiency virus
MACEs: major adverse cardiac events
PCI: percutaneous coronary intervention
